# Soft Devices for High-Resolution Neuro-Stimulation: The Interplay Between Low-Rigidity and Resolution

**DOI:** 10.3389/fmedt.2021.675744

**Published:** 2021-06-14

**Authors:** Ieva Vėbraitė, Yael Hanein

**Affiliations:** School of Electrical Engineering, Tel Aviv University, Tel Aviv, Israel

**Keywords:** neurostimulation, prosthesis, electrode substrate, electrode adhesion, multi electrode arrays

## Abstract

The field of neurostimulation has evolved over the last few decades from a crude, low-resolution approach to a highly sophisticated methodology entailing the use of state-of-the-art technologies. Neurostimulation has been tested for a growing number of neurological applications, demonstrating great promise and attracting growing attention in both academia and industry. Despite tremendous progress, long-term stability of the implants, their large dimensions, their rigidity and the methods of their introduction and anchoring to sensitive neural tissue remain challenging. The purpose of this review is to provide a concise introduction to the field of high-resolution neurostimulation from a technological perspective and to focus on opportunities stemming from developments in materials sciences and engineering to reduce device rigidity while optimizing electrode small dimensions. We discuss how these factors may contribute to smaller, lighter, softer and higher electrode density devices.

## Introduction

Neuro stimulation is used in many medical technologies, with some devices already approved for clinical use. Since its early days, the field of neurostimulation developed hand-in-hand with advances in other fields. The adoption of emerging technologies, such as silicon micro-fabrication, wireless energy transfer, hermetic packaging, application-specific integrated circuit (ASIC) technology, flexible electronics and many more, assisted in the rapid development of the field. Despite great progress, the field has not yet reached a maturation stage. In particular, high-resolution with high flexibility is a major challenge and the exploration and testing of new materials and technologies is still ongoing. The field is also challenged by limited standardization. Many research laboratories use unique material preparation protocols, which may result with dramatically different properties for the “same” material. For example, thin film deposition parameters of TiN or IrOx (to name just two examples) can result with very different porosity and material stoichiometry leading to entirely different performances. In particular, electroplating, electrochemical etching, or coating electrode sites with conducting polymers can improve electrode performances (1–5). Another common example is polymer preparation details, which may induce dramatic changes in water absorbance (as one critically important example). A related challenging issue is the use of non-standard *in-vitro* and *in-vivo* tests, which are very common in academic investigations.

In this paper, we focus on high-resolution neuro-stimulation devices. We aim to highlight the considerations that influence material selection, and discuss how these factors presently limit device performances. We aim to emphasize the key role of different materials in facilitating high-resolution neural stimulation, along with reduced device dimensions and mechanical impact. We focus on three fundamental challenges in contemporary neurostimulation devices: substrate rigidity, electrode performance and device-tissue anchoring. In particular, we highlight the role of soft polymeric materials such as PDMS, polyimide, parylene, silk and shape memory polymers as substrate alternatives. Alternative electrode materials will also be reviewed. Finally, we discuss the electrode–tissue interface, focusing on rapid and long-term device anchoring, a critically important topic, yet the least investigated thus far. We use examples based on our own work experience to highlight workable solutions to some of the challenges we present in this paper. Many other aspects (such as low power ASIC design, energy transfer approaches and wireless communication) are not discussed as they go beyond the scope of this paper (6–9).

### Brief History of Implantable Electrodes

Neurostimulation is an old practice, dating as far back as Volta's pioneering studies on electro-chemistry. Volta's efforts were followed with progressively deeper understanding of neuro-anatomy and function. In the 1930's, Hess implanted electrodes in the brain of cats, demonstrating efficacious neurostimulation. Amazingly, 70 years ago, Delgado used radio frequency-controlled wireless implanted neurostimulators in animals and humans (10). These pioneering studies were followed by refinements and improvements that resulted in highly miniaturized, multi-electrode and wireless systems.

**Figure 1:**
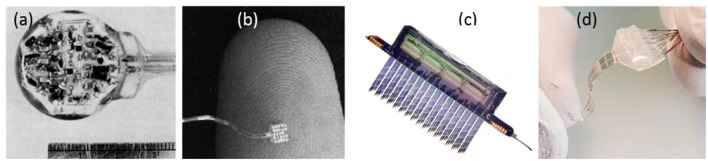
Neurostimulation devices representing 70 years of progress. **(a)** Delgado's device with RF receiver and hermetic seal (11). **(b)** The artificial retina device by Liu et al. (12). **(c)** A 256-site 3D device for simultaneous recording and stimulation in the central nervous system (13). Picture Credit: Center for Wireless Integrated Microsystems, University of Michigan. **(d)** Spinal cord stimulation device with stretching ability (14). Copyright 2015, EPFL/Alain Herzog.

Figure 1 shows four examples of neuro stimulation devices developed over the course of the last 70 years. Devices typically include active electrode sites, electrode wiring to electronic circuitry, electrical circuitry and modules for wireless communication.

In particular, silicon technology was recognized as an important enabling technology in neuronal interfacing in the late 1960's that offered excellent mechanical, electrical and later also optical properties. Wise and Najafi suggested the use of silicon technology to realize silicon shanks with high-density microelectrode arrays suitable for local recording and stimulation of neurons (15, 16). This technology, commonly referred to as the Michigan probes, has been used extensively on rodents to study the fundamentals of neuronal circuits (17–20). These devices were later further improved upon and can now boast amplification circuits (21). Norman at the University of Utah developed the Utah arrays, which consist of long and sharp penetrating silicon (22, 23). These arrays were used extensively for basic research but were also implanted in monkeys and humans (24–29). Beyond its electrical and mechanical properties, silicon also offers superb optical properties. This led to the interest of using silicon photo diodes in artificial retina devices (30). In the pioneering work of Chow and later improved by Zrenner, silicon photo diodes were implanted under the retina (31–34). Silicon photo diodes designed for the IR range were also demonstrated to be a highly effective method (35, 36).

Despite its many beneficial qualities, silicon is rigid (Young's modulus 150 GPa) compared with soft tissue (Young's modulus 0.4–15 kPa), which may cause substantial mechanical mismatch. Extensive investigations were directed in recent years to better understanding effects associated with this mismatch and developing novel soft interfaces with significantly lower mismatch and possibly better long-term performances. New electrode materials were also developed to improve device overall performances.

### Current Applications of Implantable Electrode Arrays

Neural stimulation was applied to various applications including upper/lower limb prostheses, vagus nerve stimulation, deep brain stimulation (DBS) for Parkinson's disease, epilepsy, and depression, cochlear implants, and visual prostheses (37–39). With the exception of visual implants, these medical devices build on low-resolution technology (4–22 electrodes). The two domains where high-resolution stimulation appears to be of highest value is cochlear implants and visual prostheses. Contemporary cochlear implants still rely on low electrode density affecting the resolution of the delivered auditory signal. To achieve high quality auditory perception, higher resolution devices and alternative approaches are being explored (40). The retina is one of the most demanding neural tissue with which to interface. Accordingly, extensive research efforts are directed to this application. Retinal implants aim to restore vision in patients that suffer from retinal degenerative diseases that lead to blindness. Electrical stimulation of remaining neuronal layers in the retina leads to artificial perception of vision. Electronic devices are studied as an alternative to available pharmaceutical therapies and emerging gene therapy or stem cell transplantation. Some systems reached commercialization, such as the Argus II epiretinal device (Second Sight Medical Products, CA, USA), Alpha IMS subretinal device (Retinal Implant AG, Germany) and others in clinical trials, including PRIMA (Pixium Vision S.A., Paris, France) and NR600 (Nano Retina, Herzliya, Israel). A review of retinal implants history starting from 200 years ago when the first idea of an artificial vision evolved is available in Berényi et al. (19). Additional systematic reviews detailing recent advances in retinal prosthetic research can be found in (20–24).

High density will surely benefit cochlear and retinal implant but can benefit other applications that are presently limited to low resolution. Moreover, it can contribute to closed-loop operation, which is highly desired and can dramatically improve device operation.

## Core Considerations

The range of consideration affecting device performances is wide and includes: electrode size, packaging schemes, bio-compatibility, substrate flexibility, device stability in physiological conditions, duration of use, and local heating. Here we address those topics which relate most directly to our main focus. In particular, we discuss the electrode and substrate materials. For more related discussion on packaging, biocompatibility, and multi electrode array recordings we refer the interested readers to (7, 41–44) and references therein.

### Fundamentals of Neuro-Stimulation

We begin with a concise explanation of neural stimulation and the core engineering considerations in designing and operating these devices. Neurons, the electrically active building block of the neural system, are primed to respond to external electrical stimulation. Under the appropriate conditions (i.e., amplitude, polarity, duration, frequency), neurons react to electric fields in their vicinity by firing action potentials in a manner closely resembling their response to natural neuronal signaling. The information that is then received by the brain can be controlled and guided to mimic natural processes.

Neural electrical stimulation harnesses voltage sensitive proteins in the cell membrane to illicit artificial neuronal activation (45). Generating an electrical potential at the vicinity of the electrode with a displacement current is considered safe and can be used for an extended duration without observed damage to the tissue or the electrodes (46). Stimulation pulses and their parameters must not damage the electrode or the tissue (47). Immune response, electrolysis of water, oxidation, corrosion or dissolution of the electrode could all be the result of irreversible faradaic processes (reduction and oxidation reactions at the electrode-electrolyte interface that result in new chemical species) (48, 49).

The distance between the electrode and the cell, as well as pulse features, affect stimulation efficacy and localization (50). Achieving localization is challenging, owing to variability in cell responsiveness to applied electric fields and how the electrode is coupled with the cells and their processes. To illustrate this point, Figure 1 shows an electrode with nearby cultured neurons and their activation probability at different stimulation amplitudes. The data show correlation between the stimulation amplitude and the number of activated neurons. Moreover, it is evident that some distant neurons are activated due to better coupling to the electrode by their neurites than neurons that are at much closer proximity.

In many applications, selective nerve activation is desired. Stimulation selectivity may be controlled by the amplitude of stimulation, but also by its pattern, width, inter-pulse width and frequency. A few examples in which these issues were studied are cuff electrode selective stimulation for sensory perception in humans, hd-TIME (high-density transverse intrafascicular multichannel electrode) electrode selective fiber activation in rats, and selective ON/OFF retinal ganglion cell stimulation (51–53). Selectivity can be also enhanced by the reduced electrode size and increased density. These parameters are discussed below.

### Electrode Size and Resolution

**Figure 2:**
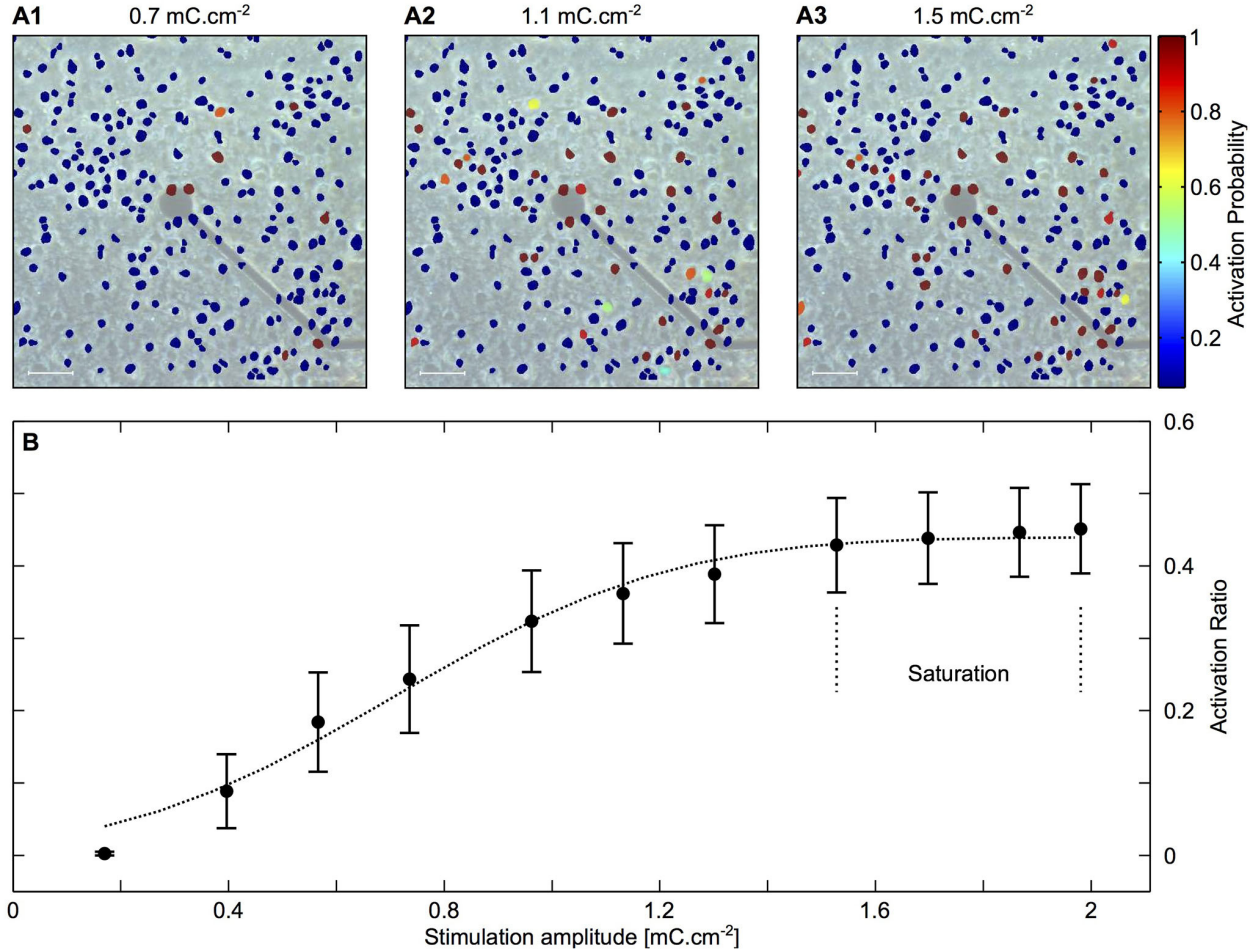
Mapping activated neurons due to electrical stimulation amplitude and location. Cortical cultures were stimulated with rectangular and biphasic 400 μs long current pulses of 25–35 μA using MEA 30 μm diameter electrodes. Neuronal action potentials evoked by an electrical stimulation were recorded and analyzed using Ca2+ imaging. **(A1–A3)** Color coded neuronal activation probability for three different stimulation amplitudes showing a correlation between stimulation amplitude and number of activated neurons. Scale bar: 50 μm. **(B)** Illustration of the latter observation showing proportion of activated neurons as a function of stimulation amplitude. The results indicate a saturation zone at which no further neurons are activated due to the distance of the electrode. Adapted from Wallach et al. (56). Copyright 2014, Wallach et al.

Each stimulating electrode has an effective stimulation range. In a simple model, this range can be considered as a uniform semi-hemisphere, which depends on the stimulation amplitude, tissue impedance and electrode area (54, 55). In reality, some cell types and regions are more sensitive than others, so an electrode may effectively stimulate distant cells while not affecting nearby units [see examples in Figure 2 (57)].

Reducing electrode size can also help in generating high-density arrays and has been the focus of many investigations. The charge injection limit determines the amount of charge an electrode can deliver without crossing the water window limit. Minimal electrode size is determined by the charge injection limit of the electrode material so the amount of charge needed for stimulation can be reached. For example, Gzahavi studied sputtered iridium oxide (SIROF) electrode surface area and charge injection properties (58). An electrode with charge injection limit of 2.1 mC/cm^2^, would have to be 95 μm^2^ in area to accommodate 2 nC needed for stimulation. See Sekirnjak et al. (55) for some typical stimulation thresholds. To facilitate charge injection increase and electrode size reduction, the electrode surface area has to be increased through surface roughening or volume increase.

Many materials were studied in recent decades as electrode material. Among the most studied are platinum, iridium oxide and titanium nitride; a detailed review of electrode materials can be found here (46, 59, 60). Some electrode-materials benefit from advantageous charge delivery capacity yet are challenged with poor compatibility with fabrication methods, in particular on soft substrates. One such example is carbon, which appears to be especially compatible for neurostimulation, yet suffers from poor compatibility with micro fabrication methods, for which reason alternative fabrication parameters and methods were investigated (61–66). Generally, metal thin films are notoriously unstable *in vivo*, suffering from dissolution, and delamination (67–71), and stable electrode material compatible with soft substrates remains a challenge. On rigid substrates, such as silicon, excellent stimulation electrodes in the diameter range of 20 μm can be realized (72). Electrode performances (both for recording and stimulation) on flexible substrate are generally inferior to those achieved on rigid substrates as the conditions required to form optimized films are less-favorable. This issue is further discussed at the concluding remarks of section Flexible Substrates.

It is important to note that thermal loading and heat dissipation should also be carefully considered in high-density applications and may limit the stimulation parameters, and consequently, the attained temporal and spatial resolution (73).

### Biocompatibilty

Upon device insertion into the body and even before stimulation, the body responds through a series of reactions. Starting with acute inflammation, a release of reactive oxidative species (ROS), followed by possible chronic inflammation. Due to ROS attack and tissue regeneration processes, implant degradation and encapsulation, which affect long-term stability and efficacy of the device, may occur (41). Therefore, device materials must be biocompatible and withstand biological reactions. Biocompatibility requirements depend on expected operation duration and environment of intended use. Medical device biocompatibility should be evaluated according to the International Organization of Standardization (ISO) standardized tests (ISO 10993) (74). Implant for neural stimulation has to show no cytotoxicity, and foreign body reaction and glial encapsulation should be mild. Bulk and surface chemical properties of a device must be carefully evaluated to guarantee its biocompatibility and stability (75). Non-fouling coatings can render a material protection against protein adhesion *in-vitro* (42). In addition, sterilization method should be carefully chosen (76). Whichever sterilization approach is chosen (i.e., ethanol sterilization, autoclaving, UV radiation, ethylene oxide gas) should not damage the electrode mechanical or optical properties, nor accelerate corrosion, denaturation or delamination of packaging materials (41, 77).

### Flexibility and Substrate Stiffness

The mechanical rigidity of neural interfaces is an extremely important property. Mechanical mismatch between the implant (Young's modulus 50–200 GPa) and the soft tissue (Young's modulus 0.2–15 kPa) may evoke an immune response, tissue scarring or trauma induced by implant placement or micro-motion of the implant, and may explain electrode degradation and reduced stimulation efficacy over time (78–82). Moreover, poor implant-tissue adhesion, vascular damage, inflammation, electrode failure, and foreign body response can be linked with device rigidity and may lead to acute and chronic responses and electrode failure (83–86).

### Device Integrity

Physiological conditions are aggressive and many materials and processes employed in the fabrication of neurostimulation devices, particularly in high-density device fabrication, poorly fit these conditions. Silicon based microfabricated and thin film processes are especially sensitive. Silicon and silicon dioxide have finite etching in physiological media (87). Stress is a major cause for failure and specially designed low-stress films had to be developed (88). Thin film deposition on polymers is particularly problematic owing to polymer swelling and films delamination.

Electrode stability under stimulation conditions is another major concern. The electrode-tissue interface is an electro-chemical interface. In the presence of ionic solution, electrode properties are determined by the nature of the electro-chemical interface that forms (89). This interface determines some of the most important aspects in neuro-stimulation. Foremost is the charge transfer mechanism, which can range from purely a displacement current for non-Faradaic interface to a one involving charge transfer (Faradaic electrodes). The electro-chemical interface also determines the impedance of the electrodes, which in turn affects the thermal noise that is picked up by the electrode when used to record electrical activity (90, 91).

To guarantee that no faradaic processes are taking place, some studies emphasize the importance of a metal-oxide passive film formation on the electrode surface, electrode passivation, that would prevent electron transport across the interface (48, 59, 90, 91). Alternatively, non-faradaic electrodes can be used to minimize charge transfer across the electrode tissue interface (in such reactions, chemical species in the electrolyte are redistributed) (92, 93). To minimize electrochemical damage, a charge-balanced biphasic waveform must be used (46, 94, 95). Other mechanisms, such as heating and electro-chemical reactions at the electrode interface, can lead to neuronal stimulation. These processes may damage the electrode or the tissue and are highly undesired. Therefore, optimal stimulation parameters, circuit passivation and device encapsulation are critical for safe stimulation, stability and biocompatibility of the device (4, 52, 94, 96).

### Acute vs. Chronic Devices and Hermetic Feedthroughs

Medical device testing is performed *ex vivo* or *in vivo* lasting several days, few weeks and up to months and years. As mentioned before, body reaction to implants occurs at the time of surgery and implant placement is considered as acute phase and response. Long-term or chronic use of medical devices is mandatory in many clinical uses. The long–term performance of the electrodes, the insulation and the packaging performance must be evaluated (77) as well as the long-term hermetic bond of the electrical feed-through connections to the electrode array. The latter is an important challenge and requires special attention. For example, in Musk (97) parylene C coated titanium case was used along with flexible probes made of gold traces encapsulated in polyimide. Parylene C serves as a moisture barrier to ensure prolong functional lifetime. In Yin et al. (98) a titanium enclosure with 100 individual hermetic feed through pins were used. The wiring to the electrodes was overmolded with silicone to establish a barrier from the ionically conductive environment. Titanium enclosures are the gold standard in hermetic sealing but different approaches were considered as alternatives. Argus II implant components (i.e., coil, electrode array, scleral band) were insulated in silicone and reached a lifetime of 26 years (in accelerated testing) (99). Doped nanocrystalline diamond channels within polycrystalline diamond insulation were also suggested as a possible solution (100). More detailed discussion of the types of hermetic sealing methods, the challenges and advances as well as non-hermetic packaging can be found in (9, 101–104).

Accelerated aging tests and hermeticity testing performed in the early stages of device development provide valuable information regarding material suitability and longevity in harsh biological environments (105, 106). Overall, preclinical studies of medical device include: (1) Acute tests—to demonstrate device efficacy. (2) Chronic passive tests—to examine biological response to implant-tissue interface and material failure. Duration varies between 1 and 12 months and depends on properties of the electrode, its biocompatibility and interface with the tissue. (3) Chronic active tests are used to evaluate stimulation safety and efficacy. Testing focuses on histological and electrophysiological changes, electrode impedance stability and stimulation performance. Long-term evaluation of device safety with large animal models is advantageous since the anatomy, surgical procedure and environment is closer to that in the clinical use (77).

### Device Anchoring

Neurostimulating devices are becoming ever more flexible, yet the electrode-tissue anchoring remains a challenge. In many reported cases, implants are mechanically attached to the soft tissue (by penetrating the tissue) or secured with sutures and/or metal tacks (107). The latter is a mature method and offers great attachment strength, but is problematic with delicate tissue, such as the retina. Even when the implant itself shows great biocompatibility, sutures and tacks can lead to post-surgical adverse effects, such as dislodging, tissue scarring and inflammation or gliosis, which may lead to reduced device function (107–111). Long-term studies found that a poorly secured implant leads to increased distance between the tissue and the electrodes, including sub-threshold stimulation (107, 112). Therefore, alternative device anchoring methods are investigated and few strategies discussed in section Device Anchoring Mechanisms and Support Materials.

### Multi Electrode Arrays

The use of micro-fabrication techniques in neuro-stimulation devices paved the way for the extensive use of multi electrode arrays (MEA) so that multiple electrodes can be simultaneously used to record and stimulate neurons. In many applications, the ability to perform both recording and stimulation at the same time and in close proximity to each other holds promise for closed loop control. The recorded signal can be used to assess the efficacy of the stimulation, which in turn can be modified to achieve a desired response (113). For example, in DBS, closed loop strategies aim to achieve higher efficiencies and possibly fewer side effects (114). Recently, Ferleger et al. demonstrated a fully implanted closed-loop DBS system for essential tremor treatment (115). High density recording may also help in source localization and noise reduction.

Having established the core considerations in designing neuro-stimulation devices, we now turn to discuss different strategies to implement flexible substrates along with high quality electrodes.

## Flexible Substrates

**Figure 3:**
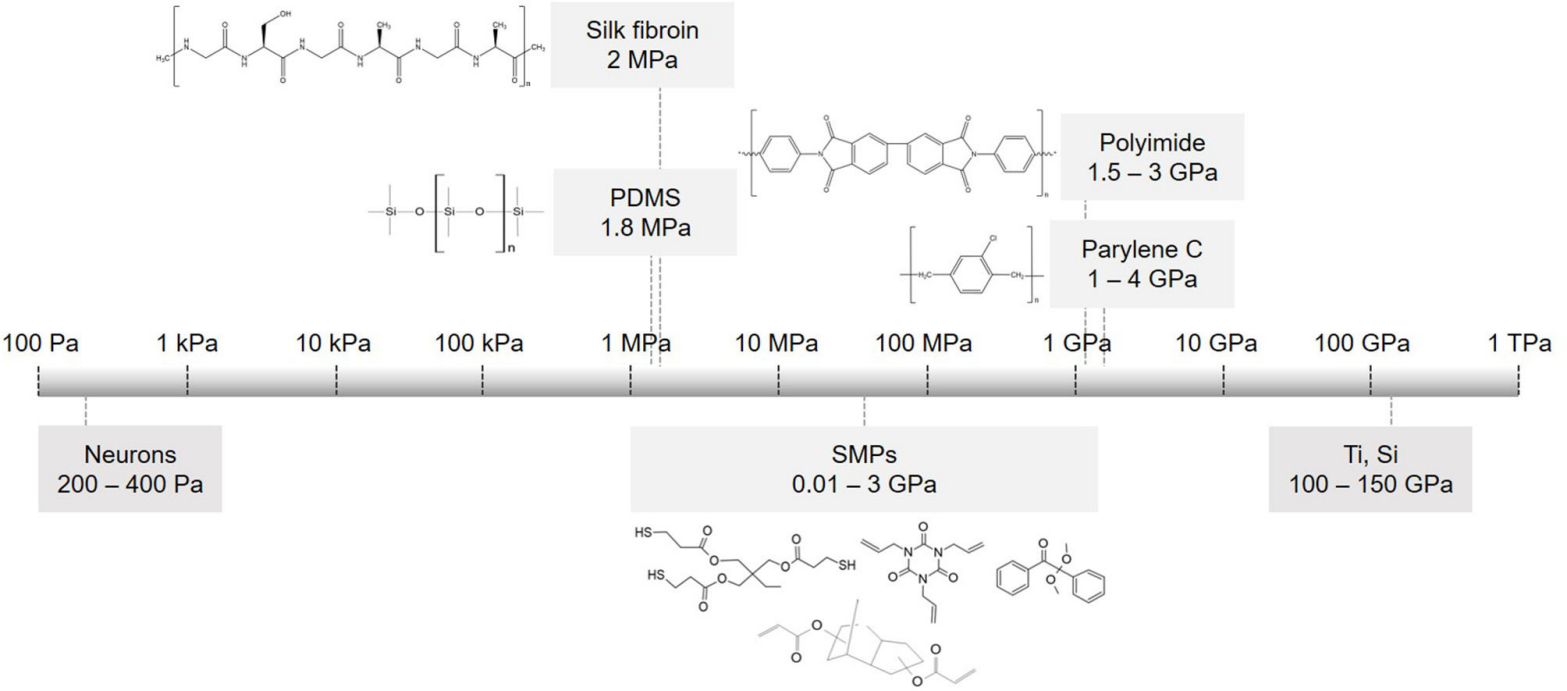
Flexible substrate materials used in implantable devices. Scale of elastic modulus for commonly used substrates and their chemical structures: PDMS, parylene C, polyimide PI 2611, silk fibroin and monomers used to make thiol-ene acrylate polymer (SMP).

The realization that the mechanical mismatch between a rigid implant and a soft neural tissue is a major factor that restricts the long-term stability of the implant led to an increased interest in soft materials (116–122). Materials such as polyimide (PI), polydimethylsiloxane (PDMS), parylene C and shape memory polymers (SMPs) (Figure 3) have significantly lower Young's modulus than silicon, hence they are gaining increased interest and are recognized as preferred substrate materials for resolving the mechanical tissue-electrode mismatch (123, 124). In addition, new materials were proposed to also substitute rigid electrode materials. Nano materials such as graphenes, carbon nanotubes and nanowires can be implemented on flexible substrates and offer marked advantages. For instance, specific capacitance values may improve from 4.5.10^−6^ mFcm^−2^ (Pt electrodes coated with SWCNT on a rigid Pyrex substrate) to 2 mFcm^−2^ (CNT on a flexible MEA) (125). These materials offer compatibility with flexible substrates along with high surface roughness and reduced impedance comparing to uncoated or gold-coated electrodes (66, 126, 127). In this section, we review soft materials used primarily as substrate materials, focusing on PDMS, polyimide, parylene C, silk fibroin and SMP separately.

### PDMS

**Figure 4:**
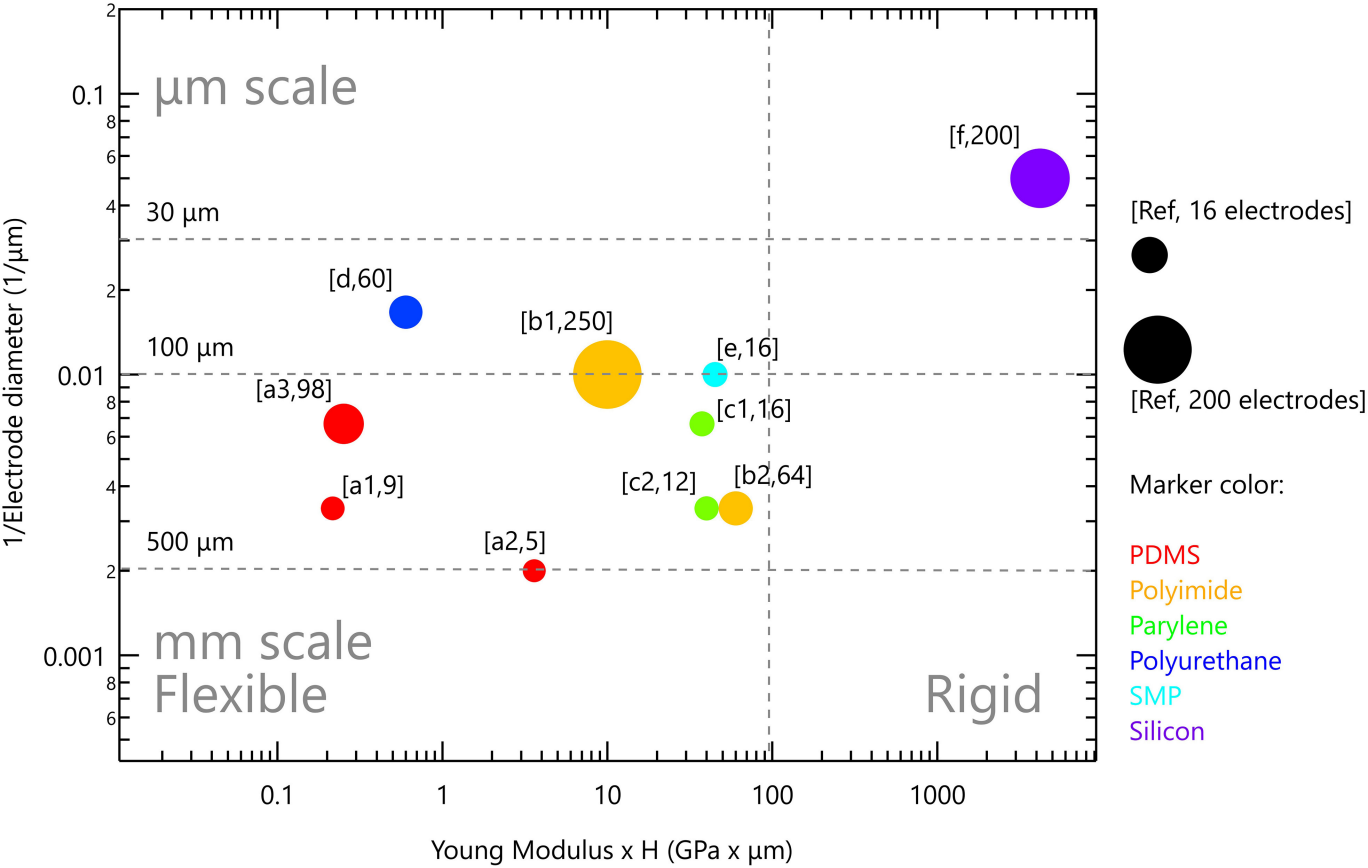
Rigid and flexible neurostimulation devices. Vertical scale is the inverse of electrode diameter; horizontal scale is Young modulus multiplied by device thickness. Marker size reflects the number of electrodes in the device. References: (PDMS) a1 (120), a2 (132), a3 (137); (polyimide) b1 (138), b2 (139); (parylene) c1 (140), c2 (141); (polyurethane) d (142); (SMP) e (143); (silicon) f (36).

Polydimethylsiloxane (PDMS), a type of silicone elastomer, has excellent mechanical properties, it is clinically approved as USP class VI and is used widely in various implants (118). It has high permeability to gases, impermeability to ions, along with optical transparency. Moreover, it has MΩ·cm resistance in its wet state (67, 128). With Young's modulus of 1.8MPa and the ability to be formed into thin films (10–100 μm), it was studied for chronically implantable devices (67). It was used as a substrate and encapsulation material in cochlear, bladder and pain controllers, to reduce the mechanical mismatch between the tissue and the device (124). For instance, when used as a substrate for peripheral nerve stimulation, it conformally wraps around the nerve and achieves a stable interface (129). EDura electrodes, with PDMS as a substrate, exhibit restored locomotion after spinal injury (120). Ferlauto et al. designed a foldable photovoltaic epiretinal prosthesis using PDMS in the shape of a dome to match the curvature of the eye (130). Hybrid electrodes for subdural neural recording and stimulation, where the electrode is based on PDMS and parylene bilayer, allowed easy handling and integration of electrodes (131). PDMS is also successfully used for electrode encapsulation for cortical stimulation and recording electrodes evoking fore- and hind-motor outputs (132). Tybrandt reported a novel inert high performance, stretchable electrode grid (SEG) for somatosensory cortex recordings. Such a device consists of Au-TiO_2_ nanowires with PDMS serving as substrate as well as encapsulation material (20). Despite its many advantageous properties, PDMS porosity is associated with swelling in wet environment which can lead to metal layer delamination, poor metal adhesion and relatively limited insulating performances (67, 131, 133, 134). In particular, metal adhesion to PDMS requires special treatment to promote stable bonding (131). Its porosity and permeability to gasses and water was recognized in the micro-fluidic community as a severe challenge and motivated studies on how to combine PDMS with other thin films without affecting its superior mechanical properties. Parylene incorporation onto PDMS suppressed water absorption and limited small molecule permeability, thus increasing its lifetime dramatically (135, 136). Several studies in the field of neuro-stimulation note that PDMS should be used with caution, even suggesting the use parylene C as a barrier or by incorporating rigid platforms below the electrodes to avoid cracks (130, 132, 137). Altogether, PDMS present use in neuro-stimulation devices is restricted to relatively thick films >60 μm, and relatively large electrodes (see Figure 4).

### Polyimide

Polyimide (PI) is an alternative flexible material extensively studied for biomedical applications. It is a polymer of imide monomers (CO-NR_2_) (Figure 3). It has been used as passivation or insulation for more than 40 years due to its excellent resistance to chemical agents, biocompatibility, low moisture absorption, high thermal stability and flexibility (Young's modulus of 1.5-3 GPa) (118, 144). Moreover, PI is compatible with standard photolithography and can be fabricated in various designs, at low thicknesses (1–15 μm) (145, 146). Polyimides are very common in neuronal interfacing devices (67, 144, 145, 147, 148). Investigation into biomedical applications focused mostly on one variant, PI-2611, that consists of biphenyl dianhydride (BPDA) and p-phenilene diamine (PPD) for their better biocompatibility and lower moisture absorption (148–150). Some neuro-stimulation devices with polyimide substrate are in clinical trials or have already been approved as safe. Retinal implant Argus II by Second Sight Ltd used polyimide as a substrate and was recently adapted for cortical stimulation (implant called Orion) (151, 152). Very recently, Neuralink announced PI-based probes for multi-site recording and stimulation from freely behaving animals for BMI applications (97). Electrodes with PI as substrate and insulating material were developed for the peripheral nervous system. Device design included sieve and cuff electrodes, and longitudinal and transverse intra-fascicular multichannel electrodes (LIFE and TIME, respectively), to achieve basic motor functions restoration (148, 149, 153–155). Various coatings like maltose, silk and polyethylene glycol were investigated to temporarily stiffen the implant during tissue penetration (156–159). Polyimide is indeed a promising material for chronic, neuro-stimulation, at least with the use of thin film electrode methods and materials, yet high-density is still limited, probably by modest electrode performances.

### Parylene C

Parylene [poly(dichloro-p-xylylene)] C, is a favorable material for encapsulation due to its insulating properties and bio-compatibility (FDA approved as USP class VI biocompatible material). Parylene C has low water permeability and moisture absorption of 0.06%. Moreover, it exhibits a low dielectric constant and loss factor; hence, it provides effective electric isolation. Parylene C can form conformal coatings as thin as hundreds of nm to ~100 μm. Young's modulus of 1–4 GPa ensures flexibility and minimizes tissue-device mechanical mismatch (67, 118, 160). Parylene was used in polyimide-based retinal prosthesis as an insulating material to improve device durability (112, 138).

Parylene is used also as a substrate material in several applications. It was shown that parylene-based implants are robust under surgical conditions and deliver efficient stimulation in *in vivo* testing (140, 161–163). Parylene is used as both substrate and insulating material for cochlear implants and cortical stimulation (141, 164). Minnikanti et al. (165) performed a comprehensive study to examine parylene C long-term stability, demonstrating that Al_2_O_3_ coating significantly enhances insulation properties and improves lifetime of neural interfaces for chronic implantation (166). Nevertheless, Oliva stresses that even though parylene C boasts excellent characteristics, specific long term biocompatibility studies must be performed for each implanted tissue (167). Moreover, parylene is susceptible to oxidation at temperatures higher than 100°C and micromachining can cause cracks, burns or wrinkles. Parylene has poor adhesion to metals, in some cases resulting in delamination and device failure in wet conditions. Post-processing techniques, such as vacuum annealing, plasma treatment, use of adhesion promoter Silane A-174, and nano-structuring the metal surface, can increase long-term stability and adhesion (76, 160, 161, 168).

### Shape Memory Polymers

Shape memory polymers (SMPs) are considered to be smart materials because of their unique ability to temporarily alter and recover their shape upon specific external stimuli, such as heat, electric field, magnetic field, or irradiation (169). As of yet, the most common group of SMPs are thermally induced SMPs, whose change in shape is initiated by a change in temperature. Such a change is the combined result of molecular polymers' network structure as well as certain programming and processing technologies. Detailed explanations of working mechanisms and shape memory phenomena can be found in (169–171). SMPs exhibit a wide Young's modulus range of 0.01–3 GPa, low density, low cytotoxicity, potential biocompatibility and biodegradability; thus, they were proposed for medical use about 20 years ago. Since then, their use and development has been continually active and growing at a rapid rate (172–174). SMPs are candidates for various medical applications from sutures to stents, from drug delivery methods to neuronal probes (175–177).

SMPs as a substrate for neural recording and stimulating electrodes were first proposed and most investigated by Voits et al. Their team reported that thiol-ene/acrylate SMPs (Figure 3) are as rigid as polyimides (about 1–2 GPa) during insertion and soften by two orders of magnitude (to ~50–20 MPa) upon exposure to physiological conditions (178, 179). They further demonstrated that these SMPs are compatible with photolithoghraphy, in wet environments take up <3% fluid, and give stable recordings *in vivo* for 2 months. Thiol-ene/acrylate SMPs showed no cytotoxicity nor neurotoxicity, and reduced the foreign body response (180). Thin film softening cuffs or spinal cord stimulation arrays provide optimal nerve-electrode interface and selective stimulation with stable long-term performance *in vivo* (143, 181). Recently, a high-density microelectrode array for retinal stimulation on SMP was developed. Upon insertion, it conforms with the eye shape (182). Zhang et al. developed 3D twinning electrodes for vagus nerve and sciatic nerve stimulation. Inside the body (at 37°C), the elastic modulus of the implant changes from 100 MPa to 300 kPa, it recovers the shape naturally and self-climbs onto the nerves to form a flexible 3D neural interface (183, 184). Compared with polyimide or parylene-C, SMPs offer reduced tissue-electrode mechanical mismatch, allowing better signal to noise ratio and reduction in stimulation thresholds. Long-term stability, robustness and device interlayer adhesion are still to be investigated (185).

### Silk

Silk fibroin is a biopolymer purified from *Bombyx mori* silkworm cocoons. The amino acid sequence of silk fibroin contains repetitive glycine-alanine-glycine-alanine-glycine-serine (GAGAGS) repeats, which self-assemble into an antiparallel b-sheet structure that gives the silk-based materials high mechanical strength (186). Silk has been used as a suture material for centuries. It can be formed into films, fibers, gels, porous scaffolds, powders, and microspheres (187–189). Silk films are non-immunogenic, mechanically flexible, show great surface quality and have optical transparency and controllable degradation rate (188–190). Furthermore, silk fibroin was shown to exhibit excellent electrical insulating properties, for which reason it is often chosen as the gate dielectric in organic thin-film transistors (191–195). Depending on application, silk films can be patterned with controllable thickness and porosity, and chemically modified with growth or adhesion factors.

Biodegradable polymeric materials such as silk gained a lot of interest, owing to their easy implantation and biodegradability. The degradation rate decreases with an increase in B-sheet content or chemical modification. These properties depend on the implantation site and the mechanical environment (196–200). Silk fibroin is a polymer candidate for tissue engineering and implantable devices (186). Rogers et al. presented gold electrode arrays for brain stimulation, with polyimide as an electrode substrate supported with degradable silk fibroin. Silk enabled conformal wrapping of the array to the brain surface (201). A parylene probe embedded in the silk to stiffen the probe for insertion into the motor cortex was also reported (202). Hronik-Tupoij et al. used silk as a substrate, and showed that electrical stimulation induced axon growth and alignment, which is critically important for peripheral nerve regeneration applications (203). A fully organic implant based on silk was demonstrated for retinal stimulation. Silk served as a substrate and photoactive conjugated polymers as a functional component (204). Silk-based flexible electrode arrays were shown to be used for localized recording and stimulation *in vivo* (205). It is indeed an attractive material in biomedical applications and is in particular preferred as sacrificial material for device insertion into the tissue. However, despite its benefits, its suitability for long-term use is limited.

Table 1 summarizes the main properties of the different substrate materials described above. Indeed, PDMS has markedly low Young's Modulus (E), yet owing to its porosity, it is used in neural stimulation applications as a thick substrate. Accordingly, its effective stiffness (EHd^3^/4L^3^ for a film with a length L, thickness H, width d and Young's modulus E) is only an order of magnitude lower than a thin parylene C. Heo et al. compared water evaporation through 8 mm-thick PDMS to 2.5 μm parylene C films (206). Because PDMS has a much higher diffusion coefficient of water (2 × 10^−9^ m^2^/s) than parylene (2.6 × 10^−13^ m^2^/s), polyimide and polyurethane, a much thicker PDMS is usually used in devices. Surface modification schemes may improve PDMS stability, but these coatings have to be validated *in vivo*. Presently, polyimide is the most studied material showing relative long-term stability *in vivo*. Flexible polymers are inherently prone to cracks and water absorption and any polymer based neural stimulation system will have to be carefully validated. Implant stiffness and thickness deserve attention as these features depend on the insertion process, the implantation site and the duration of the intended use (short- or long-term). Moreover, it is preferable to choose materials that are USP or ISO 10993 approved. Nevertheless, since each device is unique, each research group should still perform biocompatibility, stability, and efficacy tests of the intermediate and final device.

**Table 1:** A comparison between different flexible materials used as electrode array substrates.

**Material**	**Young's modulus (GPa)**	**Moisture absorption (%)**	**Diffusion coefficient (m^**2**^/s)**	**Specific resistivity (Ω·cm)**	**Dielectric Constant**	**Typical thickness (μm)**	**Typical test duration *in vivo* (weeks)**	**Regulatory**	
								**USP Class VI; ISO 10993**	**Examples for FDA/CE approved devices**
PDMS (67)	0.0018	<1–3	2*10^−9^ (206)	10^5^	2.6–3.8	120–500	4–26	MED-10xx (NuSil): ISO 10993 3–6, 10–11 (207)SILASTIC MDX4-4210 (Dow corning): ISO 10993-1; USP class VI (208)	Bladder stimulator, cardiac pacemaker.
PU (142, 209)	0.007–0.03	1.5	3.2*10^−10^ (210)	10^11^	8.8	NA	NA	TPU (Pellethane): USP Class VI (211); TPU (Texin), 9832 (3M): ISO 10993−1 (212)	Packaging
SMP (174, 178)	0.01–3	<3	NA	10^14^	NA	30–100	NA	Many tested according ISO 10993–5 (185)	For blocking blood flow
Silk (213)	0.02	NA	NA	NA	6.1	30	24		Sutures, Scaffolds, drug delivery platforms
Polyimide (67)	1.3–3	0.5	1.1*10^−10^ (214)	10^16^	2.9	7–20	2–72	“Comply with, but not ISO certified.” (150)	Retinal, Cortical implants, Pacemakers, catheters
Parylene C (67)	1–4	0.06	2.6*10^−13^ (206)	10^12^-10^16^	2.95–3.15	6–20	12	VSi: USP Class VI, ISO 10993 4–6, 10–11 (215)	Coating material

*References mentioned in the table are those that provide the most detailed information related to the addressed properties*.

Figure 4 presents the flexible neurostimulation devices we reviewed in this paper and have been tested *in-vivo*. Each device is plotted with the inverse of electrode diameter as the vertical scale and Young modulus multiplied by device thickness as the horizontal scale. Marker size reflects the number of electrodes in the device. It is clear that contemporary flexible devices have reduced electrode performances. Soft devices with μm scale electrodes, such as those available for silicon-based devices, are not yet available. It is our aim to highlight this gap, and to discuss possible directions to address it. Two strategies are discussed below: (1) Improved device anchoring to achieve better electrode-tissue coupling and (2) Improved electrode technology which will increase electrode charge injection limit and will lead to the ability to form smaller and more stable electrodes.

## Device Anchoring Mechanisms and Support Materials

Several strategies are commonly applied to anchor and stabilize devices into a tissue and were discussed in section Device Anchoring. Here, we discuss the idea of ideal adhesive for device anchoring to the tissue and alternative methods to sutures and tacks.

An ideal adhesive will allow: (1) rapid anchoring; (2) strong attachment; (3) long-term stability, non-inflammatory and non-toxicity. Various hydrogels, such as cellulose, alginate, polyvinyl alcohol or polyethylene glycol were proposed in recent years (216). Alternatively, high-surface area materials were shown to enhance the interaction with the tissue. Carbon nanotubes, in particular, have higher surface area and strong cell-electrode coupling (95, 217). Implants with apertures, allowing cell migration and anchoring of the tissue at close proximity to the electrodes were also studied (73). These solutions can improve tissue-electrode interface over time, but do not provide the rapid anchoring needed.

Bio-adhesives, such as cyanoacrylate and fibrin glue, are used to close leaking sclerotomies, to treat corneal perforations, to secure the retina after detachment, and to seal wounds. These glues offer easy application with the setting time lasting from 10 s to 2 min (218). However, the use of these bio-adhesives may result in complications such as incomplete closure, foreign body response, and viral infection. The main downside of fibrin glue includes low adhesive strength and limitation to biological implants, while cyanoacrylate offers a strong bond and adhesion to non-biological materials. Yet, under some conditions, it can be toxic and cause inflammation (109).

N-isopropyl acrylamide (NIPAM) is yet another promising bioadhesive material for implants. NIPAM is a thermo-responsive polymer that exhibits a lower critical solution temperature (LCST) of about 32°C. Below LCST, NIPAM is hydrophilic and soluble, while above LCST, it becomes a hydrophobic and viscous gel with strong adhesion to tissue. As this process is reversible, NIPAM exhibits a great advantage over other bioadhesive materials. An investigation into NIPAM properties for tissue engineering purposes, cell proliferation and adhesion, drug delivery, and intra-vitreal injections demonstrated that it is non-toxic and safe (219–221). In a comprehensive cytotoxicity study, it was shown that pNIPAM-coated surfaces are not cytotoxic, while NIPAM monomer in pure powdered form is. Thus, it was stressed that device viability depends on the purity of the polymer and the deposition type (222). pNIPAM in a liquid form was used in rabbits to close scleral wounds and resulted in effective wound healing with no abnormalities or inflammatory reactions (223). It was also studied *in vivo* in rabbit eyes for over 6 weeks. Flexible implants made of parylene C and PDMS were coated with NIPAM by plasma deposition. Once the implant was placed next to the retina, the implant was gently pressed on the retina for 15–20 s. Immediate adhesion was observed and a 6-week follow-up revealed no retinal tears nor occurrence of retinal detachment (224).

Bio-inspired materials exhibit interesting adhesive properties. The use of an active form of Vitamin B2 (riboflavin-5-phosphate) for photochemical tissue bonding showed impressive results in ocular surgery. Unfortunately, it is not suitable for anchoring devices to the retina due to ultraviolet light required to activate the bonding procedure (109, 225, 226). Other sources for bio-inspired adhesives come from marine animals, such as mussels and sandcastle worms (227–229). Spider silk is yet another material representing one of the strongest natural fibers that boasts adhesive strength in wet environments (230–232). Spider silk glues well to wood, plastics, silicone, and can be used in biomedical applications (233, 234). Cell adhesion peptides such as RGD motifs also improve device-tissue adhesion, yet will not support rapid anchoring (235, 236). Even though materials like NIPAM, RGD motifs or bio-adhesives are promising alternatives for improved anchoring, their use in neuro-stimulation devices requires further investigation.

Another important aspect that needs to be considered is implant insertion to the target tissue. Reducing the rigidity of the electrodes and loss of device stiffness makes tissue penetration challenging. This applies to applications where electrodes have to penetrate brain tissue. Various coatings like maltose, silk and polyethylene glycol (PEG) were investigated to temporarily stiffen the implant during the penetration (156–159). Another approach is the use of a guide such as a rigid shuttle device (237, 238). Apollo et al. reviewed the most recent innovations in flexible neural electrode insertion approaches, including Tyrosine-derived terpolymer, poly (vinyl alcohol) (PVA) and poly (lactic-co-glycolic acid) (PLGA), microactuation, and magnetic and bioinspired surgical implantation strategies (239).

## Electrode Materials

The conventional electrode materials, which have been shown to work very well on rigid substrates, have to be carefully optimized to reach similar performances on flexible and soft materials, specifically, the impedance and charge injection limit. Challengingly, data regarding these values is not always reported. Several emerging solutions were suggested in recent years, four of which are discussed below.

### CNTs

**Figure 5:**
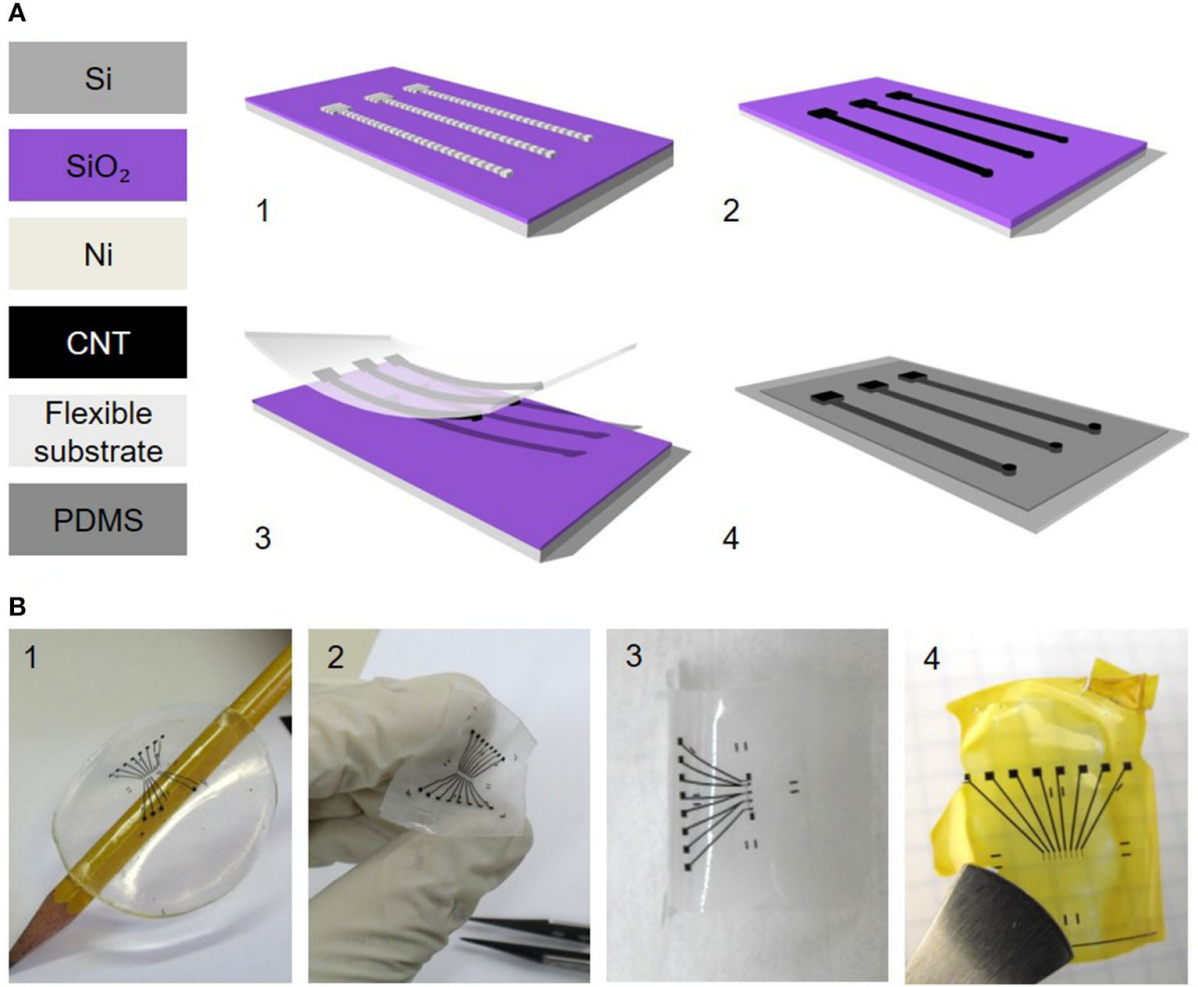
Carbon nanotube-based flexible electrodes for neuronal stimulation. **(A)** Electrode fabrication scheme. (1) Photolithographically defined Ni catalyst layer. (2) CNT film CVD growth. (3) Film transfer to a polymeric support. (4) A second polymeric layer (PDMS) with predefined holes is bonded with the CNT carrying film for passivation. **(B)** Different patterns of flexible CNT electrode arrays on different support layers: (1) PDMS, (2) medical adhesive tape, (3) parylene C and (4) polyimide (125). Adapted from David-Pur et al. (125). Copyright 2013, the Author(s).

Nano materials offer an interesting alternative to the more conventional materials. Nano electrodes with increased roughness, using nanowires, graphene, conductive polymers or carbon nanotube (CNT) coatings can resolve long-standing challenges (95, 216, 240). In particular, carbon nanotubes (241, 242) which exhibit Young's Modulus as high as 1 TPa and tensile strength of 100 GPa, can bend and twist without breaking, and are therefore an appealing material for stable, thin and flexible electrodes (243–245). CNTs are known for their utility in recording neuronal signaling, demonstrating reduced impedance and much higher signal to noise ratio (156, 217, 246–248). The *in vivo* biocompatibility of CNTs and other carbon materials was addressed by Baldrihi and Veronica, concluding that it strongly depends on the administration site, dosage, purity, and agglomeration (241, 242, 245). In 2005, it was shown that CNTs can be used for neuronal signal improvement and enhanced dendrite elongation as well as cell adhesion and growth (217, 249, 250). This was followed by a first demonstration of *in vitro* stimulation of neurons with CNT electrodes (243). One of the significant advantages of CNT electrodes is their electrical and mechanical interface with neurons. Their tridimensional structure and high surface area increases electrode capacitance, lowers the impedance, therefore enabling size reduction of the electrodes to achieve high density devices with high efficacy local stimulation, and reduces the tissue inflammatory response (240, 242, 251–253). Vitale et al. demonstrated neural recording and stimulation using CNT fiber electrodes, when neurons *in vivo* were activated as efficiently as metal electrodes with a 10 times larger surface area (254). Direct electrical stimulation of neurons by using CNT electrodes was presented by several groups (126, 253, 255), as well as extracellular stimulation using CNT MEAs (126, 243, 256–258). David-Pur et al. presented a completely flexible micro-electrode device based on various flexible substrates (PDMS, adhesive medical tape, parylene C and polyimide shown in Figure 5B) with MWCNT traces and stimulating electrodes for high efficacy neuronal stimulation (125, 259, 260). The general fabrication process is described in Figure 5A. The extraordinary strength, flexibility, surface morphology, and electrical conductivity of CNTs make them a strong candidate for neuronal interfacing in small, high-charge density and low-impedance flexible microelectronic devices. CNT-based electrodes exhibit some of the best electro-chemical performances, yet their fabrication process is non-standard and incompatible with conventional fabrication approaches. Adoption of this technology will require process automation.

### Conducting Polymers (CPs) Hydrogels (CPHs) and Elastomers (CEs)

Conductive polymers (CPs) are organic polymers possessing high electrical conductivity, mechanical softness, biocompatibility and easy surface modification. Thus, they are widely used as electrode coating materials in biomedical applications (261). Most common CPs used for neural interfacing electrodes include polypyrrole (PPy), poly(aniline) (PANI), polythiophene (PTh) and poly(ethylene dioxythio- phene) (PEDOT) (216, 262, 263). The oxidized polymer carrying a positive charge are typically doped with negatively charged counter-ions, such as poly(styrene sulfonate) (PSS) or paratoluene sulphonate (pTS) and other variations. Such CP coatings of electrode sites increase charge storage capacity, provide low impedance and high charge injection limit, thus improving tissue stimulation. Nevertheless, many reports still raise concerns about its mechanical stability in chronic implantation (264).

Studies proposed combinations of CPs and elastomers, CPs and hydrogels or CPs and CNT composites. Conducting polymer hydrogels (CPHs) result in softer films (Young's modulus of 2 MPa) while maintaining CP electrical properties. Another advantage of CPHs is its surface hydrophilicity compared to CPs (265). Need to note that with hydrogels introduce porosity and swelling, thus degree of cross-linking has to be chosen carefully. Reviews discussing chemical properties, fabrication processes, challenges and future perspectives can be found (266, 267). Integration of CPs with elastomers such as polyurethane (PU) or PDMS yield conductive elastomers (CEs). Such combinations as in CPHs maintain electrical performance of CPs and provide mechanical elasticity (142). Du et al. demonstrated that ultra-soft CE micro-wires (Young's modulus lower than 1 MPa) reduced inflammatory response and caused less distortion in an 8-week implantation period compared to tungsten electrodes (268). Also, 1 month post-implantation results showed reduced macrophage activation compared to PI implants (269). Ferrari et al. presented all polymer printed nerve cuff electrode with five PEDOT:PSS with 10% glycerol electrodes (at the final area of 130 × 130 μm^2^) (270). Yuk et al. demonstrated capability to print nine PEDOT:PSS electrodes 30 μm in diameter for *in vivo* recording of neural activities (271). Combination of CPs with other semiconducting materials such as poly(3-hexylthiophene) (P3HT) was shown to be able successfully stimulate retina up to several months (204). Nevertheless, open challenges in using these materials in high-resolution neurostimulation include their long-term stability and their ability to form high-resolution patterns with existing approaches.

### Opto-Electrical Stimulation and Photosensitive Organic Pigments

Wiring and hermetic feed-throughs is a major challenge limiting the ability to power many electrodes simultaneously (100). Therefore, techniques suitable for light directed activation of neurons are gaining interest (121, 272). Several review papers describe the various optical stimulation methods available and their challenges (273–275). In the scope of this paper, it is interesting to highlight photoelectrical stimulation, in which semiconducting films or particles absorb light to generate charge distribution equivalent to that produced by metallic electrodes (260, 273, 276–279). In particular, photo-capacitive stimulation devices based on semiconducting films share many of the considerations we discussed above.

**Figure 6:**
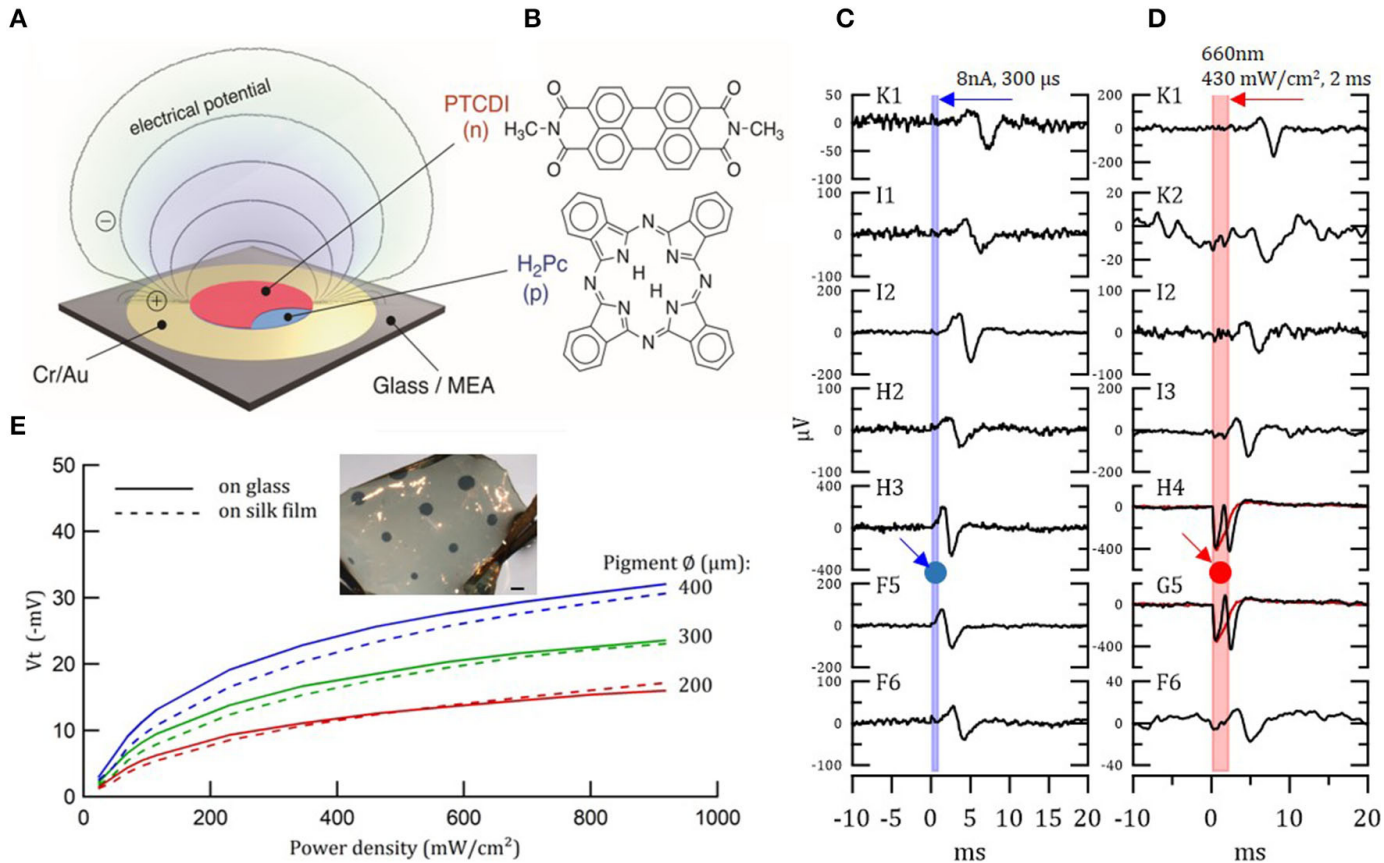
Organic photocapacitor device. **(A)** Schematic of the photocapacitor device consisting of sequentially evaporated Cr/Au and H2Pc (p-type) and PTCDI (n-type). **(B)** Molecular structures of the pigment semiconductors. **(C,D)** Action potential generation in light-insensitive chick embryo retinas in response to current **(C)** and photoelectrical **(D)** stimulation, recorded with 30 μm diameter TiN electrode MEAs. Retina was placed on the MEA/photocapacitor device with ganglion layer facing down. **(C)** Direct action potential responses in the retina to 8 μA 300 μs biphasic current pulse injected to a single electrode of the MEA (G4). Relative location of the stimulating electrode, G4, is marked by a blue arrow and circle. The graphs illustrate the latency of the response, which increases with increased distance from the stimulating electrode. **(D)** The same as in **(C)** direct action potential responses in the retina to 660 nm 430 mW/cm^2^ 2 ms photostimulation through the x40 microscope objective illuminated from above. Relative location of the illuminated photocapacitor device is marked by a red arrow and circle. Electrodes H4 and G5, which are close to the source, also recorded the electrical signal generated by the device. Reproduced with permission (280). Copyright 2018, Wiley-VCH. **(E)** Photo-electric responses of illuminated organic photocapacitor pigments on silk film vs. glass deposition. Inset—organic photocapacitor pigments deposited on a silk film. Scale bar−600 μm (281).

Photoelectrical stimulation was employed already 40 years ago with silicon to stimulate the retina. Nevertheless, silicon-based devices are rigid and alternatives flexible devices are highly desired (273). A novel photo-stimulation of neural cells was recently proposed, showing that organic pigments can transduce optical signals into electrical stimulation. Such stimulation occurs via a photocapacitive effect (280). Specifically, functional biocompatible semiconductors from hydrogen-bonded organic pigments: metal-free phthalocyanine (H2Pc) and N,N'-dimethyl perylenetracarboxylic diimide (PTCDI) were used (Figure 6B). These materials are stable in air and, in wet environments, they are biocompatible and non-toxic (282). Moreover, they can be tuned to absorb light in the 700–900 nm region. H_2_Pc absorbs light and functions as a p-type electron donor, while the PTCDI acts as n-type electron acceptor, generating a negatively-charged surface. This photo voltage buildup depolarizes the cell membrane and gives rise to an action potential. A single-, double- and triple-layer p-n device can be used for neuronal stimulation, as direct retinal responses were observed in embryonic chick retina (Figures 6A,C,D) (280). We studied the transfer of these organic photocapacitor pigments to soft silk films and compared their functionality to films formed on a glass surface. Pigments of varying sizes, ranging from 200 to 1,000 μm in diameter, were successfully deposited on silk films (Figure 6E). Voltage transients Vt–photovoltages measured above the pigment regions (Figure 6E) validate electrode functionality (281). Similarly, photoelectrical stimulation of retina via P3HT [poly(3-hexylthiophene-2.5-diyl)] with PEDOT:PSS was demonstrated by Maya-Vetencourt et al. (204). Glowacki et al. demonstrated chronic peripheral nerve stimulation via transduction of deep-red light into electrical signals for up to 3 months. Nevertheless, the electrode performance decreases over time *in vivo* (contradicting accelerated aging results), thus, the device stability and efficiency *in vivo* should be improved (283). At present, the pigment-based photocapacitor sizes are still relatively large and improved efficiency is needed to achieve the desired dimensions.

### Liquid Metals

Liquid metals are an emerging material gaining interest in applications for biosensors in wearable and implantable devices (284). Liu et al. extensively researched this field and suggest liquid metals as a preferable alternative that benefit from low mechanical mismatch and low corrosion (285). Liquid metals have a Young's modulus even lower than nerve tissue, setting them as particularly promising materials. Particularly promising are liquid metal gallium (Ga) and gallium-based alloys, which are also considered to be biologically safe. Gallium exhibits a low melting point (29.8°C) and high thermal (GaIn_20_: 26.58 Wm^−1^K^−1^ at 20°C) and electrical conductivity (2.2 × 10^6^ Sm^−1^) (286). Patterning LM can be done by lithography (lift-off), injection, additive approaches (microfluidic inkjet dispensing, stretching, selective wetting, thermal evaporation) and subtractive techniques (in-plane capillarity) (287). Guo et al. presented a flexible microelectrode array for bullfrog sciatic nerve stimulation using LM. The electrode consisted of 4 LM electrodes 500 μm in diameter on 500 μm-thick PDMS film (288). To the best of our knowledge, high-density neural stimulation devices implementing liquid metals have not been presented thus far.

## Summary

Table 2 lists several notable technologies that have been researched and developed for neural stimulation in recent years. Investigation duration, animal model and anchoring procedure vary considerably between studies, with only few studies reporting investigations of different flexible materials under otherwise similar conditions. In Minev et al. (120), 120 μm PDMS implants (EDura) were compared to 25 μm-thick polyimide in a 6-week study. Rats with EDura were indistinguishable from control while PI-implanted rats had significant motor deficits as well as significant deformation of spinal segments under the implant and neuro-inflammatory responses in the vicinity of the implant. A thinner PI implant (2.5 μm) was more conformal and exhibited less neuro-inflammatory response. A comparison between SMP- and parylene C-based arrays implanted by the spinal cord was reported in Garcia-Sandoval et al. (181). Parylene C introduced slightly more compression, but no significant tissue injury or inflammation for both arrays was observed.

**Table 2:** Flexible devices for neural stimulation.

**Materials**		**Device**	**Model and duration**	**Anchoring**	**Results of tissue response**	**Reference**
**Substrate**	**Electrodes**					
PDMS 2 mm thick	Au	Cortical stimulation	*In vivo*: rats, 10 weeks	Sutures, screws, dental acrylic	No mechanical damage; No notable foreign body response	(132)
PDMS 1.25 mm thick	Pt	Cuff around sciatic nerve	*In vivo*: mice, acute test	Electrode wrapped around the sciatic nerve	No thermal damage to the tissue	(129)
PDMS 500 μm thick	Pt/Au	Epidural spinal cord stimulation	Minipig, 6 months; Performance evaluation only;	–	Implant position might have shifted, possible build-up of scar tissue	(289)
PDMS 140 μm thick	Pt	Subretinal stimulation	Electrochemical characterization;	-	NA	(137)
PDMS	PEDOT-PEG/CNT	Tibial nerve stimulation	*In vitro*: cytotoxicity *In vivo*: rats, 1 month	Hypodermic needle shuttle for insertion	Less scar tissue encapsulation, less changes to axon size, density and morphology, reduced macrophage activation compared to polyimide implants	(269)
PDMS 120 μm thick	Pt	EDura	*In vivo*: rat, 6 weeks	Sutures, micro-screws, dental cement, surgical silicone.	Limited foreign body reaction	(120)
PDMS 64 μm thick	PEDOT:PSS/P3HT:PCBM/Ti	(nir)Polyretina	*Ex vivo* mice; No tests *in vivo*	Tacks	NA	(130, 290)
Polyimide 10 μm thick	IrOx	IRIS retinal implant (discontinued)	*In vivo*: Humans up to 30 months follow-up	Retinal tacks	Minor retinal changes, no retinal tissue damage; One patient suffered a retinal detachment during the procedure; no further adverse reactions observed during the 3-month follow-up.	(139, 291)
Polyimide 5 μm thick	Au/IrO	Retinal stimulation	*In vivo*: rabbits, 12 weeks	Retinal tack	Surgery safe but difficult; Retinal detachment, corneal edema, insufficient fixation	(138)
Photosensitive polyimide 30 μm thick	Pt	Epiretinal stimulation	*In vitro*: cytotoxicity test *In vivo*: rabbits, 6 months	Titanium tacks	Non-toxic; no local retinal toxicity; no mechanical compression	(292)
Polyimide “Thin PI film”	Pt	Epiretinal stimulation Argus II (discontinued)	*In vivo*: humans 3–6 years follow up	Scleral flap Retinal tacks	Normal inflammation; no ocular hypotony; 40% of patients experiences significant adverse effects: conjunctival erosion, hypotony, conjunctival dehiscence, presumed endophthalmitis, need for retacking; Increased expression of glial fibrillary acidic protein; fewer neurons and inflammatory reaction in the tack site	(293–295)
Polyimide 7 μm thick	Pt, coated with Pt black/IrO/PEDOT	Sciatic nerve stimulation	*In vitro*: cytotoxicity *In vivo*: rat, 2–4 weeks		Non-toxic; no significant inflammation; no rejection response; Thinner fibrous capsule developed around the implants compared to PDMS implants	(149)
Polyimide 12 μm thick	Au/Pt	Sciatic nerve stimulation	*In vivo*: rats, acute test		Minimal pressure on the nerve	(155)
Polyimide 20 μm thick	Ti, Pt, Au	Deep brain stimulation	*In vivo*: rats, 30 days	Tungsten guide to insert probe	Thin fibrosis around damaged tissue	(296)
Polyimide 10 μm thick	Pt	Tripolar spiral cuff electrode	*In vivo*: rats, 2–6 months		Very mild foreign body reaction; did not change the nerve shape; no morphological evidence of axonal loss or demyelination (except one case of partial demyelination)	(154)
Polyimide 18 μm thick	Pt	Cuff electrode	*In vivo*: rabbit, acute test		NA	(297)
Polyimide 12 μm thick	Pt black	Cuff vagus nerve stimulation	*In vitro*: cytotoxicity test *in vivo*: rats, acute test	Sutures	NA	(298)
Parylene 16–20 μm thick	Ti/Pt	Epiretinal stimulation; Spinal cord stimulation	*In vitro*: efficacy and stability test *in vivo*: canine (retinal implant), 6 months; Mice (spinal cord implant) Acute test	Sutures, tacks	No obstruction and vessel leakage	(140)
Parylene 16 μm thick	Ti/pt	Cortical stimulation	*In vivo*: rat, 12 weeks	Ti screws, dental cement	No adverse events reported; Limited tissue reaction	(141)
Parylene 6 μm thick Kapton tape as a carrier	Ti/Pt	Cochlear implant	*In vivo*: cat, acute test		NA	(164)
Parylene 5 μm thick	H_2_Pc/PTCDI	Sciatic nerve stimulation	*In vivo*: rat, 3 months	Zip-tie locking mechanism	No pathological differences between the implanted and contralateral sciatic nerve;	(283)
Silk (grooved) 90 μm thick	Pt/Ti/Au	Neural growth	*In vitro* only		NA	(203)
Silk 30 μm thick	P3HT and PEDOT:PSS	Subretinal stimulation	*In vivo*: rat, 6 months		Retina remained intact; no trophic effects	(204)
SMP 50 μm thick	TiN/Au	Spinal cord stimulation	*In vitro*: accelerated aging test *In vivo*: rats, 16 weeks	Screws and dental acrylic	Less tissue deformation than Parylene-C arrays; No significant astrogliosis or immune reaction; no noticeable neurological changes	(181)
SMP 30 μm thick	TiN/Au	Sciatic nerve stimulation	*In vitro*: compatibility test *In vivo*: rats, 30 days	Sutures, silicone elastomer	Significantly less inflammation, less fibrotic vimentin immunoreactivity compared to silicone cuff	(143)
SMP 100 μm thick	Au/Ti/PI	Vagus nerve stimulation	*In vivo*: rabbit, acute test		NA	(184)
Polyurethane (PU)	PEDOT:PSS		*In vitro*: No tests *in vivo*		Promotes neurite outgrowth, cell adhesion;	(142)

In trying to generalize the results presented in Table 2, we note the following: PDMS, parylene C and polyimide were extensively studied. PDMS investigations are based on relatively thick films and relatively short durations (several months). Parylene C often suffers from cracks limiting its durability and use to few months. PI is the most established material used also in devices approved for human investigation. A balance between flexibility and long-term stability is a key to establishing a superior substrate material. Emerging materials should be investigated that take into account these considerations and offer the desired improved qualities.

Integration of highly performing electrode materials on flexible surfaces has been demonstrated, yet performances are not yet optimized to the level achieved by rigid devices (see Figure 2). These electrode materials also must demonstrate balance between high performance and device integrity and stability. Finally, device anchoring remains a challenging issue: sutures and tacks are still the common device-anchoring mechanism. These solutions might not be sufficient, and may lead to implant displacement and tissue scarring, which would subsequently lead to a decrease in stimulation efficacy.

It is clear that, while many investigations utilize materials and systems that are presently not suitable for long-term clinical use in humans, these studies provide valuable insights toward better understanding the significance of material flexibility in neural stimulation applications.

## Discussion

While neural stimulation was indeed demonstrated long ago, achieving high resolution, low power along with safe stimulation has proven to be far less obvious (96, 299–301). Research in recent decades focused on better understanding how neurostimulation works, to guarantee the safety of the devices (capacitive behavior of the electrodes, stimulation parameters, passivation of the devices), to achieve biocompatibility (non-toxic materials, stability and biocompatibility testing), and to reduce the subsequent physiological reactions (implantation method, material stiffness, anchoring of the device) which often occur when these devices are introduced into the body, and later during continuous mechanical movements. It is important to note that immune response and adverse events vary with the location of implantation, animal model, procedural details and exact material properties (Table 2). Therefore, short- and long-term studies should be done according to the standard procedures, and in thorough comparative model.

In this review, we discussed how neurostimulation improved over the years. Commercially available cochlear and retinal implants, deep brain stimulation as well as brain-controlled prosthetics are only a few examples. Nevertheless, contemporary implants are typified by relatively high rigidity and weight, rendering them susceptible to increased risk of tissue damage, inflammation and device degradation. The main aim of current research efforts in the field is to develop devices that are substantially more adept to interface with neural tissue, to enable high-resolution, effective stimulation, accompanied by easy implantation and long-term stability. Many recent studies introduced novel materials for implantable electronics applications, including CNTs, polyimide, PDMS, parylene C and organic semiconducting pigments. These materials have the potential to overcome the drawbacks of materials used today. Implementation of novel materials in neurostimulating devices will enable further optimization; in particular, better electrodes are needed to achieve high-resolution stimulation and closed-loop operation. Finally, the need for instant and stable device anchoring needs more attention.

One of the biggest challenges in attempting to compare different materials for neuro-stimulation is the insufficient use of standardized tests. Standardized lab tests, such as cytotoxicity, are readily available at certified laboratories, yet are often performed in academic research laboratories under different testing conditions. Long-term stability and stimulation efficacy tests are performed under widely varying conditions, making systematic comparison even more challenging. Based on existing reports it is also difficult to directly compare life expectancy values. It appears for example, that the life expectancy of PDMS and Parylene C are both limited owing to inherent instability but exact values are missing. Moreover, only a few studies performed explicit comparison between materials, or used a quantitative reference (120, 181). We made an effort to highlight these issues.

Finally, it is important to note that future devices should accommodate both neuro-stimulation and recording to enable closed-loop operation, which is highly desired in many applications (302). Although recording and stimulation are closely related technologies, extensive optimization will have to be performed for each application (303–306).

## Author Contributions

YH and IV wrote the review together. All authors contributed to the article and approved the submitted version.

## Conflict of Interest

The authors declare that the research was conducted in the absence of any commercial or financial relationships that could be construed as a potential conflict of interest.
